# A questioned authority meets well-informed pregnant women – a qualitative study examining how midwives perceive their role in dietary counselling

**DOI:** 10.1186/s12884-015-0523-2

**Published:** 2015-04-10

**Authors:** Anna-Lena Wennberg, Åsa Hörnsten, Katarina Hamberg

**Affiliations:** Department of Public Health and Clinical Medicine, Family Medicine, University Hospital, SE-901 85 Umeå, Sweden; Department of Nursing, Umeå University, SE-901 87 Umeå, Sweden

**Keywords:** Antenatal care, Midwifery, Dietary counselling, Role change, Qualitative content analysis

## Abstract

**Background:**

During pregnancy and afterward, a healthy diet is beneficial for the expecting mother and her foetus. Midwives in antenatal care have an ideal position for promoting healthy diets. Dietary counselling is however complex and recommendations can be controversial. While pregnant women struggle with dietary recommendations, midwives struggle with a lack of authority. The aim of the study was therefore to describe how midwives perceive their role and their significance in dietary counselling of pregnant women.

**Methods:**

An interview study was conducted that involved twenty-one (21) experienced midwives, who worked in the Swedish prenatal health care. A qualitative content analysis was conducted.

**Results:**

Pregnant women were perceived to be well informed, but they needed guidance to interpret information on the Internet. They were described as rigorous and eager information seekers who needed guidance to interpret information as they were worried and emotional. The midwives saw themselves as a questioned authority who lacked support. This meant being informative and directive though not always updated or listened to. Their impact was uncertain and they could also lack sufficient competence to counsel in delicate issues.

**Conclusion:**

The midwives’ directive role may obstruct the women’s needs to manage the dietary recommendations and risk evaluation in a women-centred dialogue. Midwives need to acknowledge pregnant women as both well informed and skilled if they are going to develop woman-centred antenatal care. Ongoing training and self-reflection will be needed to make this change.

## Background

During pregnancy and delivery, a healthy and nutritionally balanced diet can minimize health risks for the expecting mother and her child and can have significant effects on the child’s future growth and development [1]. Many women of reproductive age only come into contact with health care during pregnancy; therefore, midwives in antenatal care have a unique role in promoting healthy behaviour and diet among pregnant women as a part of regular antenatal check-ups [2]. Pregnant women are also frequently seen as receptive to directly or electronically delivered health messages [3,4]. They use the Internet for interactions, such as communication for getting and giving support to other pregnant women; something that has the potential to empower them with respect to lifestyle changes [5]. However, a Swedish study reported that pregnant women rarely discussed information retrieved from the Internet with their midwives [6]. In a British study pregnant women requested healthy eating information early in the pregnancy, and they also wanted dietary support from women who had themselves struggled with their diet while pregnant [7].

Health literacy among pregnant women varies between countries with women in western and northern Europe reportedly having the highest literacy [8]. Health literacy has been described at three levels: functional, interactive and judgmental literacy. Functional literacy concerns knowledge of health risks and compliance with prescriptions. Interactive literacy concerns skills to extract information from different sources. Judgmental literacy concerns the analysis of information to control life events and situations [9]. Schulz & Nakamoto [10] have advocated supporting both health literacy and empowerment to enable people to take an active role in decision-making regarding their own health. This corresponds well with the concept of person-centred care, which implies taking the patient’s preferences, values, needs, and priorities into account when planning, performing, and evaluating care.

Person-centred care is described as a paradigm shift in nursing and health care and something that should be supported and implemented in all aspects of health care [11]. It implies a mutual partnership where the health professional’s medical expertise and the patient’s expertise in self-management activities in their everyday life are exchanged on an equal basis. Person-centred care is interchangeable with woman-centred care, which is a more appropriate concept for this article and will be used throughout the rest of this manuscript [12,13]. The International Confederation of Midwives (ICM) advocates that midwifery care be based on woman-centred care where midwives, in partnership with the women, empower the pregnant women to assume responsibility for both their own health and the health of their families [14].

Difficulties for health care professionals to deliver individualized dietary counselling and self-management support are reported to be a prominent barrier to changing dietary habits among pregnant women [15]. Although there is a paucity of research in the area of the role of midwives in health promotion practices such as dietary counselling, a study from the UK, reports from an interview study that even if midwives acknowledged their role in supporting health, their practice predominantly consisted of health information. Barriers were inadequate training and concerns about the midwife-woman relationship [16]. An integrative literature review of 33 research reports aimed at answering the question “What makes a good midwife” reports that good communication skills, a caring approach and individual treatment of women are essential [17].

Dietary counselling is particularly complex. Recommendations have changed over time and are sometimes controversial and scientific “facts” about risks vary between countries [18,19]. Examples are the varying recommendations about fish and cheese intake, as well as alcohol.

Pregnant women are reported to struggle with their diet [20], and midwives seem to struggle with how best to provide them with dietary information [21]. There is little evidence in the literature for how best to assist pregnant women in reducing diet-related risks while simultaneously not increasing guilt and worry about causing harm to themselves and their unborn child cf. [22]. The qualitative study in the present work was conducted in order to show how midwives perceive their potential to influence the dietary habits of pregnant women. The aim of the study was therefore to describe how midwives perceive their role and their significance in dietary counselling of pregnant women.

## Methods

This is a secondary analysis of data from previous telephone interviews complemented with new, additional face-to-face interviews. The design of using two qualitative data collection methods is labelled a mixed-/mono-method. The telephone interviews that were performed in 2012 aimed at exploring how midwives in antenatal care perceived counselling pregnant women in dietary issues. An article describing these midwives’ strategies for challenging dietary counselling situations has been published [21]. The interviews contained more data than was reported in the article, particularly about midwives’ views of pregnant women and their own significance in dietary counselling. We therefore decided to perform a secondary analysis with a new aim, which would complement the existing data with the new data from 2013. This accounts for the time interval between data collections.

### Context

The study was conducted in Sweden. Maternal health care in Sweden is provided free of charge as a part of the public sector. Most pregnant women take part in the maternal health program, which in non-complicated pregnancies entails eight to ten visits to a midwife in a primary care setting and includes health counselling about diet.

### Participants and settings

A total of 21 female midwives participated. They had between six months and 31 years (mean 13 years) work experience in antenatal care and were employed within Swedish maternal health care (Table 1). The midwives were a sample of convenience and were selected geographically in order to generate data from different parts of the country. Eleven midwives were from the northern part of Sweden, seven were from the middle part, and three were from the southern part. Fourteen of the midwives worked in urban clinics, five worked in rural clinics, and two worked in both rural and urban clinics. Recruitment was based on recommendations from local coordinating midwives and local health care managers, as well as through “snowball sampling”. Initially 22 participants were nominated and contacted over the telephone and by mail. If they were interested in participating, a time for an interview was decided upon. Five of the 22 contacted midwives declined participation and the remaining 17 agreed to a telephone interview. Face-to-face interviews with four additional participants were conducted to complement and deepen the data material. These four interviewees were, for convenience, selected from the region where the researchers were situated.

**Table 1:** **Midwives’ working areas and work experiences**

**Midwife code**	**Geographical working area**	**Working rural/central**	**Work experience as midwife**	**Work experience in antenatal care**
	**(N = North M = Mid-Sweden S = South)**	**(Rural = R/Central = C)**	**(Years)**	**(Years)**
M1	N	C	5	4
M2	N	R	2	1,5
M3	N	C	1	0,5
M4	S	C	31	22
M5	M	C	19	16
M6	N	C	23	17
M7	N	C + R	29	3
M8	M	R	26	24
M9	M	C	32	25
M10	S	C	26	16
M11	M	C	35	25
M12	N	C	38	31
M13	N	R	15	15
M14	M	C + R	2	2
M15	S	R	38	17
M16	M	R	28	13
M17	M	C	17	15
M18	N	C	13	13
M19	N	C	8	7
M20	N	C	26	10
M21	N	C	36	6
**Total**	7 N	5 Rural/	Mean 21	Mean 13
	7 M	14 Central	(Median 26)	(Median 15)
	3 S	2 Both Rural & Central		

### Interviews

The first author conducted all interviews. The 17 telephone interviews were semi-structured and were conducted in spring 2012. An interview guide was used, and the questions concerned when, what, and how dietary advice was given; challenges experienced in dietary counselling; and examples of situations where dietary counselling was perceived as successful or not. The additional four face-to-face interviews were conducted in autumn 2013 to deepen the content of the data. For these interviews, a similar interview guide was used as for the telephone interviews along with some additional questions such as “Please describe how women relate to your counselling” and “How do you perceive your influence on pregnant women’s eating habits?” Probing questions such as “Could you give an example of what you mean?” or “Please describe how you felt and thought in that moment” were asked. The initial telephone-interviews lasted 20 to 40 minutes and the additional face-to-face interviews lasted 40 to 50 minutes. The interviewer strived to create a trusting and non-judgmental atmosphere during the interviews so that the midwives would not experience the study as a means of pointing out personal failures in their counselling.

### Analysis

All interviews were transcribed verbatim. Qualitative content analysis was used for the analysis. According to Lindgren et al. [23] the qualitative content analysis comprises phenomenological descriptions of the manifest concrete content, close to the text, as well as hermeneutic interpretations of the latent abstracted message, yet still close to the subjects’ experiences. The analysis aims to highlight similarities and differences between and within meaning units, codes, categories, and themes. The analysis addresses the manifest and latent content in the text. The categories are answers to “what questions” and the themes, as used in this study, are answers to “how questions” that were asked during the analysis of the interview transcripts [24].

The analysis started with the thorough reading of the text, where after meaning units corresponding to the aim of the study were identified. When required, the meaning units were condensed and shortened but with a retained core content. The meaning units were compared and coded, i.e. given a label, and sorted into themes at different levels. All authors discussed every step of the analysis, from coding, made by two of the authors, initially together and later by the first author alone, to interpretation. The discussions continued until agreement about the content and labelling of the themes and subthemes was reached. This procedure enhanced the reliability of the findings [25].

### Ethics

The Regional Ethics Review Board approved the study (Dno 2011-426-31).

## Results

The analysis resulted in two themes, each having three subthemes (Table 2). The subthemes are described and exemplified with direct quotations from the interviews. In general, pregnant women were depicted as being well informed but too emotionally oriented to evaluate the information they received and to assess risks. Therefore, they needed midwives as a “sounding board” and for hands-on guidance. The midwives, on the other hand, described themselves as authorities, although their authority was often questioned.

**Table 2:** **Themes and subthemes describing midwives’ perceptions of pregnant women and of their own role in dietary counseling**

**Themes**	**Pregnant women: well-informed but in need of guidance**	**The midwife: a questioned authority with a lack of support**
Subthemes	Eager information seekers	Informative and direct, but with insufficient dietary knowledge
	In needs of hands-on guidance to interpret information	Listened to, but with uncertain impact
	Too worried and emotional	Inadequate competence to provide counselling in delicate situations

### Pregnant women: well informed but in need of guidance

In general, the midwives perceived most of the mothers-to-be as eager information seekers who had gained much dietary knowledge. Despite this, these women were viewed as being in need of support in evaluating, acting upon, and handling the information they had come across to assess risks. Without counselling the information exacerbated poor dietary choices among the women, e.g. not eating fish at all to avoid toxins.

#### Eager information seekers

The midwives described how almost all the pregnant women they met with were eager to learn. Many women were described as having searched for information long before their initial antenatal visits, and some were even perceived as knowing more about diet during pregnancy than the midwives themselves.

*“The pregnant women, they Google a lot, I have to say, so they are, in fact, many times almost more updated than I am.” (Midwife 9)*

The younger women were described as “living on the net” and seeking information there to become informed about dietary recommendations and how to best counteract risks for their babies. This was a situation the midwives found troublesome because they had doubts about the quality of Internet information. The midwives described how they often found dietary recommendations on the Internet to be too vague and sometimes contained contradictory and confusing messages.

*“This generation of pregnant women, they are scouring the Internet for information. But this doesn’t make things easier because a lot of the information and recommendations out there are not evidence-based.” (Midwife 7)*

#### In needs of hands-on guidance to interpret information

Most of the midwives described pregnant women as having problems sorting and interpreting the information they got from various sources, including the media, the Internet or from their mothers and friends who were not always aware of current recommendations. The women were described as having too much confidence in the advice and information they came across and by being thorough and even finicky they did not allow any deviation from the recommended diet. Some women requested detailed recommendations and wanted yes or no answers, something the midwives declined.

*“They would ask for weekly menus they could follow, but they must have some sort of common sense to manage their food intake.” (Midwife 5)*

To support dietary changes, the midwives usually tried to give clear advice and also confirm that the woman had understood the message correctly. Written recommendations from the National Food Agency (NFA) were handed out and, if necessary, this material was referred to repeatedly during later visits in antenatal care.

The midwives had difficulties knowing how to act when meeting with women with social or economic problems or symptoms of ill health related to the pregnancy because the midwives felt that dietary advice might put unnecessary extra pressure on these women. Highly educated women were also described as difficult to support. They were often seen as less susceptible to dietary advice because they were highly focused on body performance and health, however at the same time they had a liberal attitude toward raw fish and wine.

*“They also exercise intensely, these city-girls here, you know, and I mean exercising five days a week. And the sushi they just have to eat every week, which they love, and they also take a glass* [of wine] *now and again” (Midwife 11)*

#### Too worried and emotional

The midwives generally described pregnant women as being emotionally oriented and sometimes lacking rationality and as being too worried and sensitive to be able to adequately assess and evaluate behavioural risks. Many women were described as exaggerating risk-related behaviour.

*“I find that expecting mothers are quite stressed about alarming news about toxins and chemicals in food, and they have problems in thinking rationally about such warnings. They feel that according to these reports they shouldn’t be allowed to eat anything at all.” (Midwife 5)*

Pregnant women of today were generally described as belonging to a generation that feels deficient about many things, including their bodies and their behaviour and feel a significant amount of responsibility for adhering to all the dietary recommendations to which they are exposed. The pressure of always following the dietary recommendations and the anxiety of eating something prohibited were described as burdens that made the already sensitive women feel even more insecure.

*“….when the night comes, her worries start. ‘What have I eaten? How do I know that the fish really was freshly smoked?’ And then all thoughts spin on. You are really not yourself, you cannot always think rationally when you are pregnant because carrying a baby makes you think and worry a lot.” (Midwife 1)*

On the other hand, some younger women were seen as unconcerned or negligent and not taking control of their eating behaviour and weight gain.

*“Women born in the 90s…it seems that they do not really consider whether what they eat is beneficial for them or not, they can have a bag of crisps and that becomes their dinner!” (Midwife 11)*

### The midwife: a questioned authority with a lack of support

The midwives described themselves as authorities who had the “correct” information to deliver. They were commonly working on their own and were responsible for counselling pregnant women with different types of problems, not just medical issues.

#### Informative and direct, but with insufficient dietary knowledge

The midwives described themselves as being very informative with regard to lifestyle-related issues. When meeting women who did not comply or who struggled with their dietary changes, the midwives emphasized how they had to be quite tough and direct for the baby’s best interests. One midwife described how she frequently even used the word “forbidden”, which is an unusual word in the context of counselling.

*“In order to get them to understand, I point clearly with my finger at the brochure showing foods such as unpasteurized cheeses and say that they are forbidden to eat.” (Midwife 8)*

Despite the midwives’ ambitions to inform and counsel women about their diet, many felt that they were not experts in nutrition and were not always up to date on the latest nutritional recommendations. For example, risk levels related to diet were mentioned as an area where they needed more knowledge. A common theme among the midwives in this study was that they lacked the time to search for information on the Internet, and the fact that some pregnant women were more knowledgeable than they were made them feel inadequate. In addition to the recommendations from the NFA, the midwives referred to information from medical authorities as reliable sources for dietary information. Other Internet sources than the NFA were often seen as causing problems because the midwives could not evaluate these sources of information.

*“I think it is important to discourage women from finding information anywhere else because we [the health care personnel in the antenatal care] are the institution that offers the most accurate information.” (Midwife 10)*

#### Listened to, but with uncertain impact

Many of the midwives felt that they were listened to, and they were mostly satisfied with their counselling because they felt that their patients usually expressed gratitude for getting “expert” advice.

*“Midwives are quite informative, and I think it’s great when they* [pregnant women] *are receptive and listen to what I say. It is also important that the fathers participate. This way, things that the woman had not understood could have been noted by the father.” (Midwife 8)*

Immigrant women were depicted as having much confidence in midwives. However, the impact of the midwives’ advice was seen as hard to evaluate, and they knew that far from all women understood everything.

*“Then we have these women from other cultures who are somewhat different than Swedish women. In one way, they believe more in what you tell them, and I emphasize that this is important. It is not certain that they adhere to what I tell them, but they do not dispute or question my advice, which is very pleasant.” (Midwife 21)*

Those with the most need, such as illiterate, socially deprived, and overweight women were considered as the hardest to counsel. The difficulties were not always due to language barriers, as cultural disparities could also present problems.

*“Even if you reach them with words, they continue to use a lot of sugar when they are suffering from nausea because they are used to it and feel that it is good for them.” (Midwife 11)*

The midwives also met many pregnant women who were uninterested or at least did not express any need for guidance, and such situations were experienced as problematic. The midwives often informed the women anyway, even if for no other reason than to fulfil expectations from authorities and guidelines.

*“Even if they are not listening or bothering, you choose to continue to inform and inform because it is easier to do so.” (Midwife 8)*

The midwives often felt that their advice had an uncertain impact on the women’s behaviour. They felt that they were sometimes too overwhelming in trying to influence the women to change their behaviour, and this was something that could lead to women distancing themselves from the midwives despite the midwives’ best intentions.

*“Sometimes I try to emphasize my opinion and guide them even if they don’t agree. However, if the intention is good, this can be excused…but sometimes you are nagging on and on without any response.” (Midwife 5)*

#### Inadequate competence to provide counselling in delicate situations

Lifestyle issues related to eating behaviour were described by the midwives as difficult and delicate to counsel, and dietary counselling was described as “walking through a minefield”.

*“The most difficult thing in dietary counselling is to avoid insulting them and hurting their feelings if they are overweight…it really is a balancing act.” (Midwife 2)*

Obese women or those who put on too much weight, as well as women who were underweight or who had eating disorders, were described as particularly challenging to counsel. The weight issue was described as “delicate” and problematic to bring up during the counselling sessions.

*“Many of those who are obese have never been asked about what their increased weight might be due to. That someone dares to talk about it is a way to show that you care about them, and this can often help the women come to grips with underlying issues involved in their dietary choices.” (Midwife 9)*

Dietary patterns were also seen as hard to change because the pregnant women still had to eat, but they had to change *how* and *what* they eat. The midwives even stated that counselling about alcohol intake and smoking was easier because recommendations for drugs and tobacco were “zero-tolerance”. Many of the midwives had taken short courses in motivational interviewing (MI), but they found that even if this is a promising counselling method continual training and self-reflection would be needed to be confident enough to put it into practice.

Most midwives stated that even if dietary counselling is a common task in their work with pregnant women, they had too little knowledge and training for this task. Instead of relying on specific counselling methods such as MI, they described how they used common sense to handle difficult situations. They also admitted that they often had no solutions for diet-related issues.

*“Obviously, because I have many patients who gain too much weight during their pregnancies, I can’t really say that I have the solution. I’m only human after all and make mistakes now and then.” (Midwife 19)*

Some of the midwives described how many difficulties were related to their own approach in counselling and suggested alternative methods to improve communication and counselling.

*“I really think that we must change our methods and become more woman-centred and more of a sounding board.” (Midwife 9)*

## Discussion

Our results showed that the midwives felt that they were being listened to, but were uncertain what impact their counselling had on the pregnant women’s behaviour. Their authority, therefore, was both ambiguous and questioned. The midwives viewed the pregnant women as eager information seekers who scoured the Internet for dietary information. However, the women were in need of guidance because they were considered to be too emotional and worried about interpreting the information and managing their diet on their own. The midwives were doubtful about the use of information sources on the Internet because they could not control or evaluate the information.

Pregnancy has traditionally been considered in midwifery as a normal life event. Nowadays, an increasing focus in midwifery care has been placed on risks and disease prevention instead of health promotion [26]. In a Swedish observational study, the midwives in antenatal care emphasized pregnancy as being a healthy condition, but at the same time they used the antenatal visits to check the pregnancies for deviations and complications [27]. Moreover, the midwives in our study seemed to medicalize dietary issues and prohibited intake of food items that might risk containing toxins or contaminants. They expected the pregnant women to follow their advice and to take responsibility for their diet while at the same time remaining relaxed and not too rigorous in relation to dietary issues. This is not an easy balancing act. Pregnant women receive advice and restrictions in the name of safety and risk-reduction, and they should certainly avoid an array of foods, but they should also avoid many other risky behaviours such as changing the cat litter and should take other precautions in their daily lives “just in case” [22]. Medicine in Western society plays an increasingly important role in shaping the ways we think about and treat our bodies. Thus medical advice influences many women to be more careful, but it can also lead to worry cf. [28]; and worrying about being a bad mother is reported to be a significant problem among many pregnant women [20].

The midwives in our study viewed themselves as experts who should provide important knowledge about risks because the pregnant mothers were seen as lacking the ability to interpret the information they found on their own. Pregnant women were even seen as enfeebled during pregnancy and, therefore, to be in need of some degree of governance for the sake of their own and their unborn child’s health. Rather than counselling, the activity could be labelled as a transfer of information about dietary change. Unfortunately, the transfer of knowledge and information did not solve the difficulties of reaching women who were described as uninterested or non-adherent, i.e., women who were obese, underweight, or living in socioeconomic or cultural circumstances that they could not easily influence. In a previous study, we reported that when dictating and governing strategies did not work, the last step was resigning responsibility and leaving the pregnant women on their own [21]. Increasing weight-gain, overweight and obesity in pregnancy is a growing health problem among pregnant women: The midwives described such counselling situations as delicate and they requested more training and education. Midwives in the UK as well as in Australia have reported a similar lack of training and education, particularly in dietary counselling of obese women. Building a trusting and supporting relationship between midwives and obese pregnant women has in previous studies been reported essential for effective care. An important issue is to identify and address possible underlying causes of unhealthy diet if they should be solved, but simultaneously avoiding communication styles that negatively impact the midwife-women relationship [29,30].

Despite that pregnant women in Europe are reported to have high health literacy [8] the midwives in our study described pregnant women as being in need of hands-on guidance to interpret health information, since they did not fully trust the women’s ability to make good judgments. Sometimes they even tried to stop them from seeking information on their own. Despite their own awareness of not having sufficient dietary knowledge, they defended their choices of prioritizing their own expertise over that of their patients and of the one-way flow of written as well as verbal information that included permissions and prohibitions based on the recommendations of the NFA. Midwives in the UK were also reported to predominately provide information instead of counselling pregnant women [16]. We have interpreted that, by holding on to one source of information and excluding or disqualifying others that they were not familiar with, the midwives in our study could more easily maintain their authority cf. [31]. Midwives in another Swedish study [32] perceived that more enquiring and knowledgeable parents undermined their professional expertise and competency as well as their control.

A traditional, authoritarian counselling style might possibly be a way to increase *functional* health literacy among pregnant women, but their *procedural* and *judgmental* health literacy will be less supported. There is the risk with such a method that there will be little opportunity for the women to develop the skills to extract information from different sources. Furthermore it could negatively impact pregnant women’s ability to critically analyze and use information to control life events and various situations that are related to dietary choices [10].

Despite midwives’ strong professional identity [32] it seems to be prime time for a professional role change from a guidance-cooperation model to a woman-centred care model cf. [10,12]. Such care derives from a mutual participation model and implies that both parts trust each other and are respectful of the other’s expectations and values cf. [33]. In a woman-centred care model, risk communication is a two-way process. In this process, the pregnant woman who actively seeks information on risks from many different sources is one important part [20,34]. The other part is the midwife, who should be skilled in counselling methods but also knowledgeable in questions about risks and risk magnitude. Alaszewski [34] problematizes the communication of risk knowledge and states that even in an area where there is scientific consensus, such as abstinence from alcohol and caffeine consumption during pregnancy, there are often alternative views. Alaszewski argues that epidemiological knowledge of the probability of harmful events occurring within populations does not address individual patients’ needs for information about their own personal risks, and it is not possible to talk about one single truth. Midwives in the UK have reported that they predominantly focus on risk assessment and health information instead of supporting women to change behaviour [16]. Also in our interviews, the pregnant women’s exposure to risks was described as a common issue in dietary counselling. However, the magnitudes of the risks were never discussed in terms of how risky a particular behaviour might be, such as eating smoked fish twice a week. Lyerly et al. [22] state that the boundaries between “dangerous” and “safe” and between “reckless” and “responsible” in pregnancy are constructed in a rigid, yet often arbitrary, manner.

Trust is a central component in risk communication and lifestyle counselling during pregnancy. It is expected that a trusting relationship will allow the midwife to be seen as a credible source of information where the midwife with whom the pregnant woman has hopefully developed a relationship cf. [34]. However, the midwives in our study seemed to question the credibility of their own advice and its impact. The medicalization of pregnancy implies an increased power of the health care professionals through monitoring and medical procedures along with excessive emphasis on medical outcomes and risk prevention [35,36]. While midwives are a part of this monitoring, risk reducing, and controlling care, at the same time they are expected to build trust and support pregnant women in developing health literacy and empowerment so that these women become more responsible for their own health choices. From interviews with Swedish midwives, Larsson et al. [32] report two existing midwifery cultures that clash and lead to decreased confidence in the professional group. On the one hand, there exists a culture of making judgements by themselves without always thinking ‘to be on the safe side’ and on the other hand they are controlled by safety and the use of medical technology and measurements. Midwives have to combine a risk-focused approach with a woman-centred approach in their counselling. If not reflected on, these conflicting priorities could become very difficult to handle and will most certainly lead to role ambiguities [37-40] or risks for burn out symptoms [32].

In the organization of antenatal care, the midwives have an intermediate role where they are expected to autonomously counsel women and monitor pregnancies, while at the same time they are closely controlled by health care organizations and government authorities through registrations, statistics, and time restrictions cf. [16]. Lack of training, insufficient knowledge, and limited time have previously been described by midwives as barriers to efficient health counselling cf. [16]. In order to fulfil governmental and institutional expectations, many of the interviewed midwives informed all pregnant women about dietary issues even if they were not in need of such information or were not interested in it. In many situations, the midwives felt insufficient and on their own in the organization and as just being solely workers, burdened and without support, only doing what they were expected to do. According to Street & Epstein [33], health service is organized in a quite fragmented manner, and thereby midwives are likely to withdraw from building relationships and instead focus solely on task completion as a result of role ambiguities [32].

### Methodological discussion

We observed when we conducted the secondary analysis of the initially collected data that the two qualitative data collection methods, labelled a mixed-/mono-method where the new, additional interviews had a slightly different focus, complemented the aim well [41].

The use of different interview techniques has also been questioned [42,43], but mixed data could be seen as a useful strategy by providing more opportunities to get answers to the research questions [42]. We did not find any significant differences when comparing telephone and face-to-face-interviews, but the four additional interviews contributed with further examples and variations. One might think that face-to-face interviews will give richer and more personal data but that was not our experience. In this study we found that the telephone interviews sometimes gave richer and also more in-depth material than the face-to-face interviews. We suggest that the telephone interviews facilitated a sort of soliloquizing by the midwives and that the “distance” allowed for more honest descriptions.

## Conclusion

The interviewed midwives viewed themselves as authorities with expert knowledge of antenatal care, but with lacking skills in dietary issues and competence when counselling for delicate issues such as weight-related problems. Pregnant women were seen as eager information seekers, something that was seen as problematic. They were also judged as needing the midwives’ guidance to interpret the information and manage their diet on their own. The midwives’ informative and directive role may obstruct the women’s needs to evaluate and manage the dietary recommendations and risk evaluation in a women-centred dialogue. Midwives need to acknowledge pregnant women as both well informed and skilled if they are going to develop woman-centred antenatal care. To address midwives’ needs for more knowledge and competence in dietary counselling it is important for health authorities to offer midwives education about healthy diet as well as opportunities for them to develop their competence in dietary counselling, for example by giving possibilities to participate in tutor led reflection groups together with other midwives and to collaborate with dieticians. Further research is needed on the interaction between the midwife and the pregnant woman and her partner in order to evaluate and increase knowledge about counselling.
